# *In-vitro *Evaluation of Protective Effects on DNA Damage and Antioxidative Activities of *Ilex Spinigera *Loes. Extracts

**Published:** 2016

**Authors:** Maryam Mohadjerani, Mina Vosoghi Roodgar

**Affiliations:** *Department of Molecular and Cell Biology, Faculty of Basic Sciences, University of Mazandaran, Babolsar, Iran.*

**Keywords:** *Ilex spinigera*, Antioxidant activity, DPPH, DNA damage, PAB assay

## Abstract

*Ilex spinigera *(Aquifoliaceae) plant is an evergreen tree or shrub with thick glossy dark green leaves and red fruits. This plant has medicinal properties and has been used traditionally in northern Iran for malaria treatment. The aim of this work is to evaluate the antioxidative activities and the inhibitory effect of *I. spinigera *on the oxidation of DNA. We have found no reports about the popular use of *I. spinigera *in terms of its chemistry and biology. In this study we report the antioxidant activity of* I. spinigera *extracts for the first time. Water, ethanol and methanol were used as extraction solvents. Various experimental models including iron (III) reducing power, total antioxidant capacity, DPPH radical scavenging activity, PAB assay and *in-vitro* inhibition of AAPH-induced oxidation of DNA were used for characterization of antioxidant activity of the extracts. The three extracts showed varying degrees of efficacy in each assay in a dose-dependent manner. The aqueous extract with the highest content of total phenolics, was the most potent antioxidant in all assays except in DPPH assay. The methanol extract with the highest amount of total flavonoids was the potent scavenger of DPPH radical with an IC_50_ value of 102.22 ± 0.001 μg/mL. Aqueous extract of* I. spinigera* also showed the protective effect on DNA damage-induced by AAPH. According to our results, *I. spinigera* leaves extract have the potential for chemoprotective studies.

## Introduction

Reactive oxygen species (ROS) such as superoxide anions, hydroxyl radicals and hydrogen peroxide are a class of highly reactive molecules that are often generated as byproducts of biological reactions or from exogenous factors (1, 2). Free radicals usually have short-lived species but they possess a single unpaired electron (3). Therefore, they play important roles in oxidative stress that is the consequence of an imbalance between many prooxidant and antioxidant factors (4). The imbalance between production and consumption of reactive oxygen species, leading to oxidative stress, is implicated in the pathophysiology of a plethora of genetic and acquired disorders, such as arteriosclerosis, Alzheimer^'^s disease, Parkinson's disease, cancer, rheumatoid arthritis and malaria, as well as aging processes and neurodegenerative diseases (1, 5).

Despite the ultimate antioxidant in plasma, immune system alone can't eliminate free radicals. In the body there is a need to provide an external source of antioxidant such as food and medicinal plants (6). Antioxidants are compounds that inhibit or delay the oxidation of important molecules by inhibiting of oxidizing chain reactions and have coevolved with aerobic metabolism to counteract oxidative damage from ROS (7, 8). Among the synthetic antioxidant types, the most frequently use to preserve in foods are butylated hydroxylanisole (BHA) and butylated hydroxytolune (BHT), propyl gallate (PG) and tert-butyl hydroquinone (TBHQ) and has led to notable adverse effects in consumer health (9, 10). Therefore, this is important to search natural antioxidants, especially of plant origin, as these show antioxidant as well as antimicrobial activities (11-13). Natural antioxidants especially of medicinal plants are associated with lower risk of dangerous disease as diabetes, cancer and cardiovascular disease (14, 15).

At low or moderate concentrations, ROS exert beneficial effects on cellular responses but at high levels, free radicals can damage biologically important macromolecules including DNA, proteins and membrane lipids (16). The mechanism for DNA damage, leading to mutation, is explained by the attack of ROS on modification at DNA bases, strand break, DNA-protein cross-link and base-free site therefore oxidative damage of DNA is regarded as the etiology of some disease (17-19).

Antioxidants mop up the excess amount of the prooxidant before they damage the essential molecules of the body. Determination of the prooxidant–antioxidant balance (PAB assay) in based on 3, 3′, 5, 5′-Tetramethylbenzidine (TMB) and its cation, used as a redox indicator participating in two simultaneous reactions (4, 20). In this study PAB value of the various extracts of *I.spinigera* was measured. 

The Aquifoliaceae is a large linage of the earliest diverging order. The present Aquifoliaceae consists of the single genus Ilex (21). The Ilex genus comprises about 450 species which are naturally distributed in tropical regions (Asia and South America) and in temperate zones in temperate zones as well (22)*. I. paraguariensis *leaves which grows in Paraguay in northern Argentina and southern Brazil, due to caffeine compounds such as coffee and tea used as tail, due to the presence of tannins, its leaves are used medicinally (23). *I. spinigera *with similar *I. paraguariensis *species apparently has not significant difference. *Ilex spinigera (*Loes.) Loes. plant is an evergreen tree or shrub with thick glossy dark green leaves and globular red fruits. This plant has medicinal properties, but has no caffeine and has been used traditionally in northern Iran (named “Khass” in Persian) for malaria treatment (24). 

During the different stages of the industrial process, the antioxidant activity of extracts from *I. paraguariensis *was characterized for the first time by two different methodologies including the DPPH free radical scavenging assay and the ferric thiocyanate assay. The fresh leaves and stems of *I.p. *are employed to prepare a tea-like beverage and are used also as antirheumatic and to treat gastrointestinal disorders for its eupeptic and choleretic properties (5). There are an increasing number of *I.p.* products patents, as well as a growing interest in its product by countries whose population do not traditionally consume beverages of* I. p. *For this reason, *I. paraguariensis *green leaves could provide the most useful material with antioxidant properties to be used as vegetal drug or extracts in different formulations for food and phytopharmaceutical and cosmeceutic preparations (5).

According to the Flora Iranica, *I. spinigera* plant is native to Iran forests, particularly, Gilan, Golestan, Mazandaran and Azerbaijan talesh area (24). In the present study we have evaluated antioxidant activities of various extracts of *Ilex spinigera *(Loes.) Loes. and their protective effect on AAPH [2, 2'-azobis (2-amidineopropane hydrochloride)] induced DNA damage. The oxidative damage of DNA induced by AAPH causes carbonyl species, that can be determined quantitatively by visible spectrum after reacting with thiobarbituric acid (TBA) to form thiobarbituric acid reactive substance (TBARS) (25).

## Experimental

*Plant material and extracts*

In this study, the leaves of *Ilex spinigera* were collected from Mazandaran forests in September 2012 and authenticated by Dr. Alireza Naghinezhad. The voucher specimen was deposited at the Herbarium of the Department of Biology, University of Mazandaran (voucher Nr.4034). Extraction was carried out by maceration method. The leaves were cleaned and dried at room temperature in Laboratory and then in an oven at 60 °C for 24 h after which the dried plant material was ground to powder using an electric mill to obtain uniform size particles. Two gs of plant sample were extracted with 3 × 50 mL of methanol, ethanol and distilled water as solvent on shaker at room temperature. Each extract was centrifuged and filtered through Whatman No. 1 filter paper. The filtrate was evaporated to dryness in vacuo at 40 °C in a rotavapor. The dried sample of each extract was weighed to determine the yield of soluble constituents and stored at 4 °C until use. 

*Chemicals and reagents*

1, 1-diphenyl-2-picrylhydrazyl radical (DPPH˙), ascorbic acid (AA), quercetin, gallic acid, folin-ciocalteu reagent, trichloroacetic acid (TCA), butylated hydroxyl toluene (BHT), ammonium molybdate, aluminium chloride, potassium ferricyanide (K_3_[Fe(CN)_6_]), and TMB (3, 3′, 5, 5′-Tetramethylbenzidine), HRP-enzyme, chloramine T trihydrate, hydrogen peroxide (30%), Tris-Cl buffer, Triton X100, Sodium dodecyl sulfate (SDS), MgCl_2_, AAPH [2, 2'-azobis (2-amidinopropane hydrochloride)], TBA (Thiobarbituric acid), n-butanol, PBS [Na_2_HPO_4_ (8.1 mM), NaH_2_PO_4_ (1.9 mM), EDTA (10.0 μM)] and all chemicals were of analytical grade and purchased either by Fluka, Sigma or Merck Co.

*Determination of total phenol and flavonoid contents*

Polyphenols (compounds that generally characterized with the presence of several hydroxyl groups attached to a ring or multi-ring structures) are classified as phenolic acids, stilbenes, lignans and flavonoids are the major plant compounds with antioxidant activity (26). Therefore, it is important to measure their amount in natural extracts. The amount of total phenolic compounds was determined according to Folin-Ciocalteu method (27). This assay was carried out following the same method as reported previously. A calibration curve of gallic acid was prepared, and the result for the extracts was expressed as µg GAE (Gallic acid equivalents/100 g extract) (28). Aluminum chloride colorimetric method was used for determination of flavonoid contents of *I.spinigera* extracts. Total flavonoid contents were calculated as quercetin from a calibration curve. The calibration curve was prepared by preparing quercetin solutions as concentrations 5-50 µg/mL (29).

*Antioxidant activity determination by DPPH radical scavenging assay*

The ability of the extracts to scavenge DPPH radicals was determined according to the method of Ardestani (30). 1 mL of a 1 mM methanolic solution of DPPH˙ was mixed with 3 mL of each extract solution in methanol (containing 25-200 µg/µl of dried extract). The mixture was then vortexed vigorously and left for 30 min at room temperature in the dark. The absorbance was measured at 517 nm and anti-DPPH activity was expressed as percentage DPPH scavenging relative to control using the following equation:

$\mathrm{DPPH˙ scavenging activity\%}=\frac{\begin{array}{c} A_{\mathrm{control}}-A_{\mathrm{sample}} \end{array}}{A_{\mathrm{control}}}\times 100$

Where A_control_ was the absorbance of the blank tube and A_sample_ was the absorbance of *I.s.* or standard. Ascorbic acid and BHT were used as positive controls. The DPPH solution without sample solution was used as a control. Triplicate samples were run for each set and averaged. The results were given as mean values ± SD.

*Total* *antioxidant capacity*

The antioxidant activity of the extracts was evaluated by the phosphomolybdenum method according to the procedure of Prieto (31). This assay is based on the reduction of Mo (VI) to Mo (V) by the sample and the subsequent formation of a green phosphate/Mo (V) complex at acidic pH. In this test different concentrations of each extracts (10-400 μg/μL) were used. An aliquot of 0.1 mL of sample solution was combined with 1mL of reagent solution (0.6 M sulphuric acid, 28 mM sodium phosphate, and 4 mM ammonium molybdate). The tubes were capped and incubated in a water bath at 95 °C for 90 min. After the samples were cooled to room temperature, the absorbance of each test tube was measured at 695 nm.

*Antioxidant activity determination by* *Reducing Power*

The reducing power of extracts was determined according to the method of Yildirim (32). Different amounts of each extracts (25-800 μg/μL) in 1 mL of distilled water were mixed with phosphate buffer (2.5 mL, 0.2 M, pH 6.6) and potassium ferricyanide [K_3_Fe(CN)_6_] (2.5 mL, 1%). The mixture was incubated at 50^ o^C for 20 min. A portion (2.5 mL) of trichloroacetic acid (10%) was added to the mixture to stop the reaction. The upper layer of the solutions (2.5 mL) was mixed with 2.5 mL of distilled water and FeCl_3_ (0.5 mL, 0.1%), and the absorbance was measured at 700 nm. Increased absorbance of the reaction mixture indicated increased reducing power of the extracts. Ascorbic acid was used as positive control.

*Protective effect on DNA damage-induced by AAPH*

*DNA extraction from Cow Blood*

DNA was extracted from cow blood using general phenol-chloroform extraction method (33, 34). The extracted total DNA was measured by the following equation: [dsDNA] = OD_260 _× 50 × 1/Dilution and estimated its molecular weight by electrophoresis on an agarose gel (1%) at Voltage 90 V for 2 h. The concentration of DNA was expressed as the millig of DNA per milliliter of PBS. The DNA bands were stained with 1μg/mL ethidium bromide and visualized by UV-transilluminator. Banding was photographed using a Gel-doc system.

*The antioxidant effect of I.s. extracts on* *AAPH-Induced oxidation of DNA*

AAPH-induced oxidation of DNA was performed according to the Zhao *et al.* method with a slight modification (17, 35). Briefly, various concentrations of plant extracts in DMSO were added to PBS solutions of AAPH and DNA, in which the final concentration of DNA and AAPH was kept 2.0 mg/mL and 40 mM, respectively. Then, the extracts were dispatched into test tubes with a final volume of 2.0 mL. Then the tubes were incubated in a water bath at 37 ˚C to initiate the oxidation. Three tubes were taken out at appropriate interval and cooled immediately, to which 1.0 mL of TBA (1.00 g TBA and 0.40 g NaOH dissolved in 100 mL PBS) and 1.0 mL of trichloroacetic acid aqueous solution 3% was added. The tubes were heated in a boiling water bath for 15 min. After cooling, 1.5 mL of n-butanol was added and shaken vigorously to extract TBARS. The absorbance of n-butanol layer was measured by a spectrophotometer at 535 nm. Different concentrations of ascorbic acid were used as positive controls. 

*Prooxidants–antioxidants balance (PAB) assay*

A modified PAB assay was applied according to the method of Alamdari (4). Sixty mg TMB powder was dissolved in 10 mL DMSO; for preparation of TMB cation, 400 μL of TMB / DMSO was added in 20 mL of acetate buffer( 0.05 M; pH 4.5), and then 70 μL of fresh chloramine T (100 mM) solution was added into this 20 mL, mixed well, incubated for 2 h at room temperature in a dark place; 25U of peroxidase enzyme solution was added into 20 mL TMB cation, dispensed in 1 mL and put at -20 °C; in order to prepare the TMB solution 200 μL of TMB / DMSO was added into 10 mL of acetate buffer [0.05 M buffer, pH 5.8]; the working solution was prepared by mixing 1 mL TMB cation with 10 mL of TMB solution, incubated for 2 min at room temperature in a dark place and immediately used. 10 μL of each sample with various concentration (0.03-3.25 μg/μL), standard or blank (distilled water) were mixed with 200 μL of working solution in uncoated micro titer plate, which was then incubated in a dark place at 37 °C for 12 min. The reaction was stopped by the addition of the stop solution (100 μL of 2N HCl) was added to each well the absorbance and measured in an ELISA reader (Biotek ELX800) at 450 nm. A standard curve was provided from the values relative to the standard solution. The values of the PAB are expressed as percentage of hydrogen peroxide in the standard solution. The standard solutions were prepared by mixing varying proportions (0-100%) of 250 μM hydrogen peroxide with 3 mM uric acid (in 10 mM NaOH). The values of unknown samples were then calculated based on the values obtained from the above standard curve. 

*Statistical analysis*

*In-vitro* experimental results were given as mean ± standard deviation of three parallel measurements. The experimental values were evaluated by using one-way analyses of variance. *P* values < 0.05 were regarded as significant.

## Results and Discussion

The antioxidant activity of phenolic compounds, as major antioxidants in plants, depends on their chemical structure, the polarity of the extracting solvents, the isolation procedures, as well as the test systems. The antioxidant ability of a compound is different according to different antioxidant assays or, for the same assay when the polarity of the medium varies, since the interaction of the antioxidant with other compounds plays an important role in the activity (9). 

The aim of the current study was to compare the antioxidant activity of *I. spinigera *extracts in three different solvent systems. The influence of the extraction solvent (water, methanol and ethanol) on the total phenolic content in the extracts and their antioxidant activity were investigated. To our knowledge, there are no reports that detail the antioxidant activity of the various extracts from the *I. spinigera* leaves. To explore the antioxidant potential of different extracts, the use of a single test is insufficient to identify the different mechanisms involved. Therefore, five antioxidant assays, namely the DPPH radical scavenging activity, total antioxidant activity, reducing power, DNA protection ability and pro-oxidant-antioxidant balance assay were performed. 

*Total phenolic (TPC) and Total flavonoid contents (TFC)*

The yield of extraction in three solvents and amounts of TPC and TFC of the leaves extracts are listed in Table 1. As shown in Table 1, the highest amount of extracted components of leaves was in water extract (13.63%, w/w) while the lowest amount of components was in ethanol extract (10%, w/w). Then water was found more effective as solvent for extraction of the plant components.

Total phenol contents, as determined by Folin Ciocalteu method, are reported as gallic acid equivalents by reference to standard curve (y = 0.0402x, r^2 ^= 0.998). The total flavonoid contents are reported as mg quercetin equivalents by reference to standard curve (y = 0.0213x, r^2 ^= 0.988). In our investigation, the highest amount of total phenolics was detected in the aqueous extract of *I.s.* (11.7 ± 0.6 mg gallic acid equivalent of phenol content in 100 g of dried extract of *I.s.*) and the methanolic extract has a highest amount of total flavonoid content (3.30 ± 0.5 mg quercetin equivalent/ 100 g of dried extract). Phenols and polyphenolic compounds, such as flavonoids, are widely found in food products derived from plant sources, and they have been shown to possess significant antioxidant activities.

**Table 1:** Total phenol and flavonoid contents of water, methanol and ethanol leaves extracts of *I.* *spinigera*.

*I.s .*extract in	Extraction yield yield (w/w %)	Total phenolics *		Total flavonoids**
Water	13.65	11.75 ± 0.6	1.30 ± 0.2	
Methanol	10.35	4.22 ± 0.4	3.30 ± 0.5	
Ethanol	10.00	3.75 ± 0.6	2.62 ± 0.6	

Values are means of three determinations ± standard deviation.

* mg gallic acid equivalent/100 g of dried extract.

** mg quercetin equivalent/100 g of dried extract.

*DPPH free radical scavenging activity*

**Figure 1:**
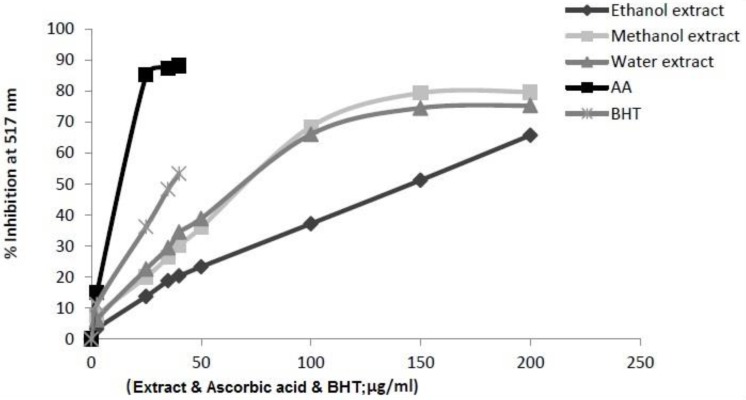
Scavenging effect of the *Ilex spinigera* leaves extracts (in ethanol, methanol and water) on DPPH radical at different concentrations. Each value represents a mean ± SD (n = 3). BHT and ascorbic acid (AA) were used as positive controls

DPPH method is economic, simple, rapid, and widely used to investigate the free radical-scavenging activities of phenolic compounds and evaluate the antioxidative activity of compounds in natural products. In the DPPH radical scavenging assay, the extracts showed a dose-dependent moderate DPPH radical scavenging activity. The ability of the extracts to scavenge of DPPH radical (IC_50_) was evaluated for AA, BHT and *I.s.* extracts. Figure 1 illustrates a significant decrease in the concentration of DPPH radical due to the scavenging ability of *I.s.* extracts and standards. AA and BHT were used as reference radical scavengers. The scavenging effect of *I.s. *and standards on the DPPH radical decreased in the following order: AA > BHT > methanol extract > aqueous extract > ethanol extract of *I.s.*, which were 17.67 ± 0.004 µg/mL, 33.90 ± 0.005 µg/mL, 102.22 ± 0.001 µg/mL, 106.58 ± 0.002 µg/mL, 145.09 ± 0.001 µg/mL as IC_50_ , respectively. Usually, higher total phenol and flavonoid contents lead to better DPPH-scavenging activity. The methanol extract of *I. spinigera* leaves with high level of phenolic contents and highest amount of flavonoids showed the best activity.

*Total antioxidant activity*

**Figure 2:**
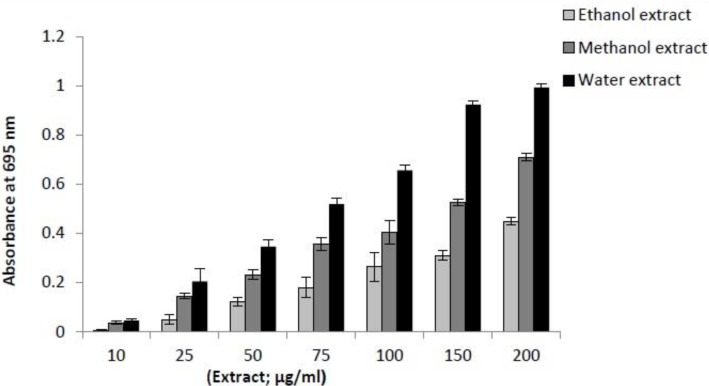
Comparison of total antioxidant activity of three extracts of *I. Spinigera *leaves (in ethanol, methanol and water) at different concentrations. Values are means of triplicate determination ± standard deviation

This assay is based on the reduction of Mo^+6^ to Mo^+5^ by reductants (antioxidants) with the formation of green phosphate/Mo (V) complex at acidic pH, which shows a maximum absorbance at 695 nm. As given in Figure 2, the results suggested that the antioxidant activities of three extracts are concentration dependent. In this study the aqueous extract showed the strongest antioxidant activity than other extracts. This could be due to the different concentrations and type of antioxidative compounds present in these extracts.

*Reducing power*

**Figure 3:**
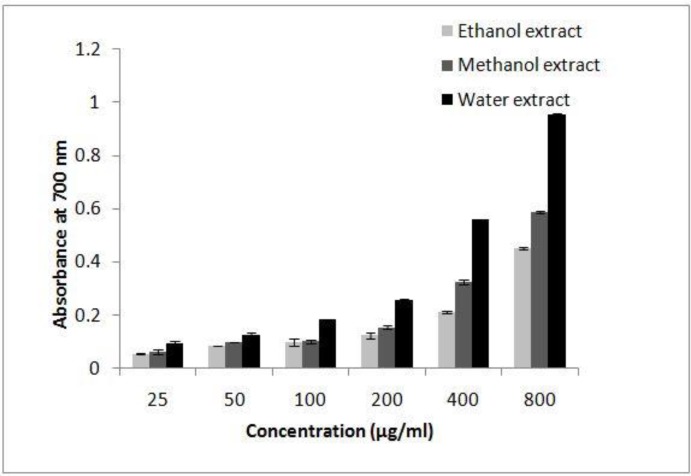
Reducing power of the three extracts of *Ilex spinigera* leaves at different concentrations, as measured by means of spectrophotometric detection of the Fe^3+^-Fe^2+^. Each value represents a mean value ± SD (n = 3).

Fe (III) reduction is often used as an indicator of electron donating activity, which is an important mechanism of phenolic antioxidant action. The presence of reductants (antioxidants) in the extracts of *I.s. *would result in the reducing of Fe^+3^ to Fe^+2^ by donating an electron. Amount of Fe^+2^ complexes can be monitored by measuring the formation of Prussian blue at 700 nm. Like the antioxidant activity, the reducing power of* I.s.* increased with increasing concentration of the sample. The results show that the reducing power was dependent on concentration (Figure 3). The reductive ability of *I.s.* extracts and standard compounds exhibited the following order: AA > BHT > aqueous extract > methanol extract > ethanol extract.

*Inhibition of AAPH-Induced oxidation of DNA*

**Figure 4:**
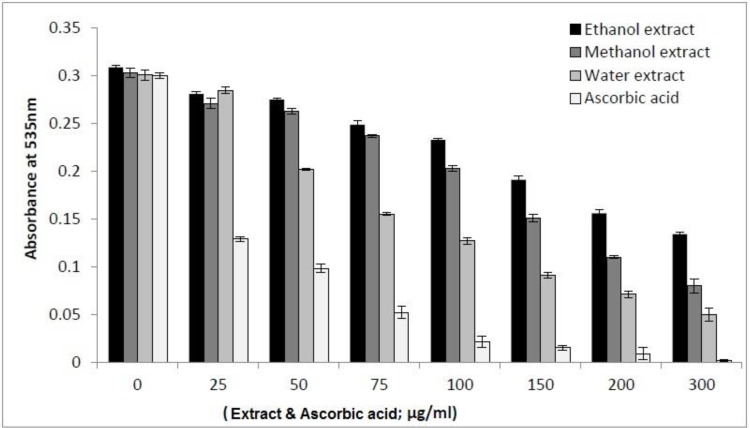
DNA protective effect of the extracts of *Ilex spinigera*. The extracted DNA from cow blood (2 mg/mL) was subjected to 40 mM AAPH-induced oxidation in the presence of *I.s.* leaves extracts at different concentrations (0-300 µg/mL). The DNA protection was measured by means of spectrophotometric detection of the TBARS in test at 535 nm. Each value represents a mean value ± SD (n = 3). Ascorbic acid was used as pure antioxidant

Several diseases are caused by damage to DNA. Thus, research on DNA protection is important nowadays. The free radicals derived from the decomposition of AAPH are able to convert the supercoiled DNA strand into open circular, linear form and carbonyl species eventually. The carbonyl species can be measured by visible spectrum after reacting with thiobarbituric acid (TBA) to form thiobarbituric acid reactive substance (TBARS) (36, 25). As shown in Figure 4, the decrease of the absorbance of TBARS at higher concentrations of the extracts indicates that the oxidized DNA diminished. The measured protective effects of *I. spinigera* extracts against AAPH-induced DNA damage exhibited the following order: aqueous extract > methanol extract > ethanol extract.

*Prooxidants–antioxidants balance (PAB) assay*

**Figure 5.**
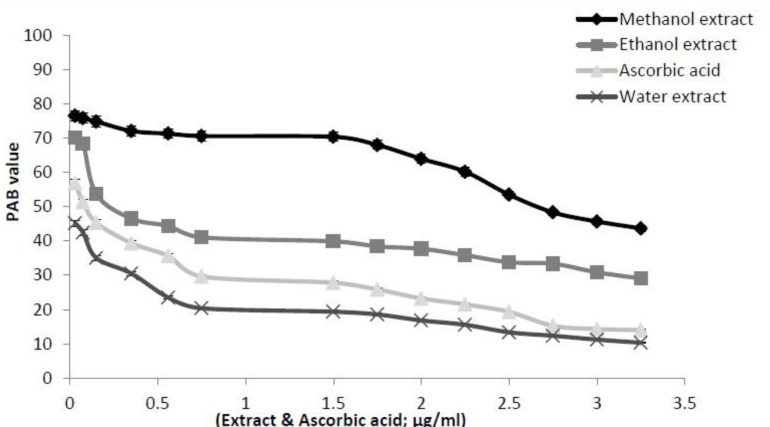
The decrease of the PAB value for ascorbic acid as standard and its comparison with *I.* *spinigera* leaves extracts (in methanol, ethanol and water) at various concentrations. The values of PAB were expressed as the percentage of hydrogen peroxide in the standard solution. The PAB values of the extracts were then calculated based on the values obtained from the above standard curve. The experiments were performed as described in “Material and Methods

In this assay TMB and TMB cation were used to measure the balance between POX and AO in plant samples. A representative standard curve was constructed by mixing varying proportions (0–100%) of Hydrogen peroxide (as a representative of the oxidants), with uric acid (as a representative of the antioxidants). It should be noted that hydrogen peroxide and uric acid do not directly interact with each other. Accordingly, PAB assay has been calibrated using a series of mixture of hydrogen peroxide and uric acid. The results are expressed as the percentage of hydrogen peroxide in the calibration mixture and PAB value shows the oxidative stress index. The standard curve shows when the percentage of oxidant is higher, the absorbance is greater. Therefore, this property can be used to determine the balance of antioxidants and oxidant in *I. spinigera* extracts. As Figure 5 shows with increasing concentration of ascorbic acid, the amount of PAB value was reduced, also with increasing concentration of *I. spinigera* extracts, the amount of PAB value was dropped. There is an inverse relationship between the concentration of extracts and amount of prooxidant material in the samples. In the PAB assay, prooxidant can convert TMB to TMB cation with blue color. Therefore, whatever the amount of prooxidant is greater, absorption increases and the amount of inhibition is reduced. So with increasing concentration of extracts due to its antioxidant properties, TMB cation converts to TMB and the PAB value reduces. According to Figure 5, the slope of descending of PAB result for the aqueous extract is higher than ascorbic acid and other *I. spinigera* extracts and follows as: aqueous extract > AA > ethanol extract > methanol extract.

## Conclusion

In conclusion, the results of our study showed the phenolic content of the aqueous extract is the greatest among the other extracts of *I. spinigera*. On the other hand, this extract showed the best results on total antioxidant activity, reducing power, prooxidant–antioxidant balance (PAB) and AAPH-induced oxidation of DNA. It can be concluded that the aqueous extract of *I. spinigera* leaves is a rich source of phenolic and antioxidant compounds. Our study has shown the methanolic extract of *I. spinigera *contained some flavonoids and showed moderate-high free radical scavenging activity when tested by DPPH˙ scavenging method. But generally the highest antioxidant activity was measured in the aqueous extract. So it is recommended that *in-vivo* tests and more concerning about these extracts of *I. spinigera*. Polyphenols and xanthines, followed by purine alkaloids, flavonoids, amino acids, minerals and vitamins might be responsible for these activities as reported in a very similar work on *I. paraguariensis *(37). 

The results of the present study indicated that polar solvent extracts are powerful scavengers of free radicals and reducing agents and therefore can be utilized as effective and source of natural antioxidants. However, further investigation is needed to identify individual compounds forming antioxidative system and develop their application as food and pharmaceuticals.
